# Knowledge, Attitudes, and Practices of Non-ophthalmologist Physicians and Optometrists Regarding Diabetic Retinopathy: A Scoping Review

**DOI:** 10.7759/cureus.105133

**Published:** 2026-03-12

**Authors:** Paula Alejandra Navarro, Carlos Mario Rangel, Virgilio Galvis, Julian Cala-Duran, Sarah Camacho, Alejandro Tello

**Affiliations:** 1 Ophthalmology, Autonomous University of Bucaramanga (UNAB), Floridablanda, COL; 2 Ophthalmology, Santander Ophthalmological Foundation (FOSCAL), Floridablanca, COL; 3 Ophthalmology, Autonomous University of Bucaramanga (UNAB), Floridablanca, COL; 4 Research, Development, and Technological Innovation, Santander Ophthalmological Foundation (FOSCAL), Bucaramanga, COL

**Keywords:** diabetic retinopathy, health knowledge attitudes practices, optometrists, physicians, scoping review

## Abstract

Knowledge, attitudes, and practices (KAP) of non-ophthalmologist physicians and optometrists are crucial for early detection and timely referral of patients with diabetes mellitus (DM). This scoping review aims to systematically map the available evidence on KAP related to diabetic retinopathy (DR) among these healthcare professionals. A scoping review was conducted following PRISMA-ScR guidelines. Electronic databases (MEDLINE, Embase, Cochrane Library, and LILACS), gray literature (medRxiv), and reference lists of included studies were searched. Two independent reviewers performed study selection and data extraction. Descriptive synthesis, evidence mapping, and summary tables were utilized to summarize findings. Seventeen studies from 10 countries, including a total of 2,627 participants, were included. The proportion of clinicians performing fundus examinations ranged from 1.3% to 54%. Knowledge of the recommended timing of DR screening in patients with type 1 DM was low (2.3%-66%), but high for type 2 DM (53%-85.5%). Common barriers to DR screening included limited confidence in fundoscopic examination, insufficient training, lack of equipment, and concerns regarding the use of mydriatics. In conclusion, substantial KAP gaps were identified across professional groups and healthcare settings. Significant deficiencies in DR-related KAP among non-ophthalmologist physicians and optometrists may limit early detection and appropriate referral. These findings underscore the need for targeted educational interventions and system-level strategies to enhance DR screening capacity in primary care.

## Introduction and background

Diabetic retinopathy (DR) is one of the leading causes of preventable blindness worldwide and represents a major public health challenge among individuals with diabetes mellitus (DM). Despite advances in screening programs and therapeutic interventions, DR remains a significant cause of vision impairment in working-age adults, particularly in low- and middle-income countries [1,2]. With the global prevalence of DM projected to increase substantially in the coming decades, the burden of DR is expected to rise accordingly, underscoring the urgent need for effective prevention, early detection, and timely referral strategies [2].

DR is a diabetes-related microvascular complication characterized by progressive retinal vascular damage that may lead to irreversible vision loss if not detected and managed promptly [3,4]. Early identification and appropriate treatment are critical to reducing vision-related disability, improving quality of life, and decreasing diabetes-associated morbidity and mortality [5]. However, the effectiveness of DR prevention strategies depends not only on the availability of evidence-based clinical practice guidelines but also on the capacity of healthcare systems and providers to implement them consistently and appropriately [6].

Non-ophthalmologist physicians and optometrists play a pivotal role in the care pathway of patients with DM. As frontline providers and often the first point of contact within the healthcare system, they are responsible for identifying at-risk patients, recommending screening, and ensuring timely referral to ophthalmology services [6,7]. Strengthening this workforce is essential to improving early detection rates and optimizing referral patterns. These efforts align with global initiatives aimed at reducing preventable blindness and achieving targets related to noncommunicable disease control [7].

Despite the existence of well-established screening guidelines, many patients continue to present with advanced stages of DR [8,9]. This gap suggests potential deficiencies not only at the system level but also in provider-level factors. The Knowledge, Attitudes, and Practices (KAP) framework is commonly used in health research to evaluate healthcare providers’ understanding of a condition (knowledge), their perceptions and beliefs toward it (attitudes), and their actual clinical behaviors (practices). Assessing KAP allows identification of educational gaps, behavioral barriers, and implementation challenges that may contribute to suboptimal screening and referral [10]. Previous studies have reported variability in DR-related KAP among non-ophthalmologist healthcare providers, particularly in resource-limited settings [6,11,12].

Given the critical role of non-ophthalmologist physicians and optometrists in DR prevention, a comprehensive synthesis of the existing evidence is warranted. Therefore, this scoping review aims to systematically map and summarize the literature on the knowledge, attitudes, and practices of non-ophthalmologist physicians and optometrists regarding diabetic retinopathy screening and referral, and to identify gaps that may inform future educational and policy interventions.

## Review

Material and methods

Study Design

In accordance with the PRISMA Extension for Scoping Reviews (PRISMA-ScR) guidelines, this study was conducted as a scoping review with methodological rigor and transparent reporting [13].

Protocol Registration

The research protocol was prospectively registered on the Open Science Framework (OSF) Registries platform [14].

Information Sources 

A comprehensive literature search of the electronic databases: MEDLINE [via Ovid Technologies Database Platform (OVID)], Embase (via Elsevier), Cochrane Library (via OVID), and LILACS [via Virtual Health Library (BVS)]. Additionally, grey literature was reviewed in medRxiv, and the reference lists of all studies included in the studies for inclusion in this review were manually checked to identify other relevant publications.

*Search Strategy* 

In July 2025, the selected electronic databases were searched. The search strategy was medical subject headings (MeSH) and free-text terms as related to physicians (general practitioners, family physicians, internists, endocrinologists, pediatricians), optometrists, diabetic retinopathy, health knowledge, attitudes, and practice. Using Boolean operators (“AND” and “OR”), the search terms were combined. No restriction was attached to publication date or language. The total search approach for the main databases is described in Table 1.

**Table 1:** Search strategy

N	Medline and Cochrane library (OVID)	EMBASE (ELSEVIER)
1	Exp Physicians/	'Physicians '/exp
2	Exp Endocrinologists/	'Endocrinologists '/exp
3	Exp General Practitioners/	'General Practitioners '/exp
4	Exp Pediatricians/	'Pediatricians '/exp
5	Exp Physicians, Family/	'Physicians, Family '/exp
6	Exp Physicians, Primary Care/	'Physicians, Primary Care '/exp
7	Exp Optometrists/	'Optometrists '/exp
8	(Physicians).tw.	(Physician*):ab,ti
9	(Endocrinologists).tw.	(Endocrinologist*):ab,ti
10	(General Practitioners).tw.	(General Practitioner*):ab,ti
11	(Physicians, Family).tw.	(Physicians, Family):ab,ti
12	(Physicians, Primary Care).tw.	(Physicians, Primary Care):ab,ti
13	(Optometrists).tw.	(Optometrist*):ab,ti
14	OR/1-13	
15	exp Diabetic Retinopathy/	'Diabetic Retinopathy '/exp
16	((diabet$ or proliferative or non-proliferative) adj4 retinopath$).tw.	(diabet* or proliferative or non-proliferative):ab,ti
17	(sleep* adj3 behavior).tw.	(sleep* NEAR/3 behavior ):ab,ti
18	diabetic retinopathy.kw.	(diabetic retinopathy):ab,ti
19	(diabet$ adj3 (eye$ or vision or visual$ or sight$)).tw.	(Diabete* NEAR/3 (eye or vision or visual or sight)):ab,ti
20	(retinopath$ adj3 (eye$ or vision or visual$ or sight$)).tw.	(retinopath* NEAR/3 (eye or vision or visual or sight)):ab,ti
21	(DR adj3 (eye$ or vision or visual$ or sight$)).tw.	(DR NEAR/3 (eye or vision or visual or sight)):ab,ti
22	OR/15-21	
23	(“Knowledge”).tw.	(Knowledge):ab,ti
24	(“attitudes”).tw.	(attitudes):ab,ti
25	(“Practice”).tw.	(Practice):ab,ti
26	(KAP).tw.	(KAP):ab,ti
27	Exp Health Knowledge, Attitudes, Practice/	'Health Knowledge, Attitudes, Practice '/exp
28	OR/ 23-227	
29	14 AND 22 AND 28	

Eligibility Criteria

These included observational studies, whether descriptive or analytical, whether or not they were available for publication assessment, that assessed the knowledge, attitudes, and/or practices about diabetic retinopathy in non-ophthalmologist physicians and/or optometrists. Studies were excluded if they were clinical trials, case reports, narrative reviews, systematic reviews, or if they focused solely on patients with DM or healthcare professionals who were not involved in the care of patients with diabetes.

Selection of Sources of Evidence 

The titles and abstracts were independently screened by two reviewers to identify potentially eligible studies. The eligibility of studies was verified, and full-text articles were evaluated. Discrepancies were resolved by discussion, and a third reviewer was called upon when needed. When further information or clarification was needed, the corresponding authors were contacted. Screening was conducted with the Rayyan Web-based program [15].

Data Extraction

The data extraction was carried out individually by two auditors on a Microsoft Excel (Microsoft Corporation, Redmond, USA) template standard sheet for documenting the data. The findings included the author's name, year published, country, study design, population details, sample size, the nature of the questionnaire, the KAP domains that participants scored, and the key findings.

Data Synthesis

The descriptive method was used to synthesize the extracted data. The key features of the included studies are summarized in tabular order. A narrative synthesis was performed to compare the evidence across professional groups and medical environments. A digital evidence map was generated using the EviAtlas application, an open-source tool, enabling visualization of the distribution of the literature and the design of the included studies [16].

Results 

Search Results and Study Selection

**Figure 1:**
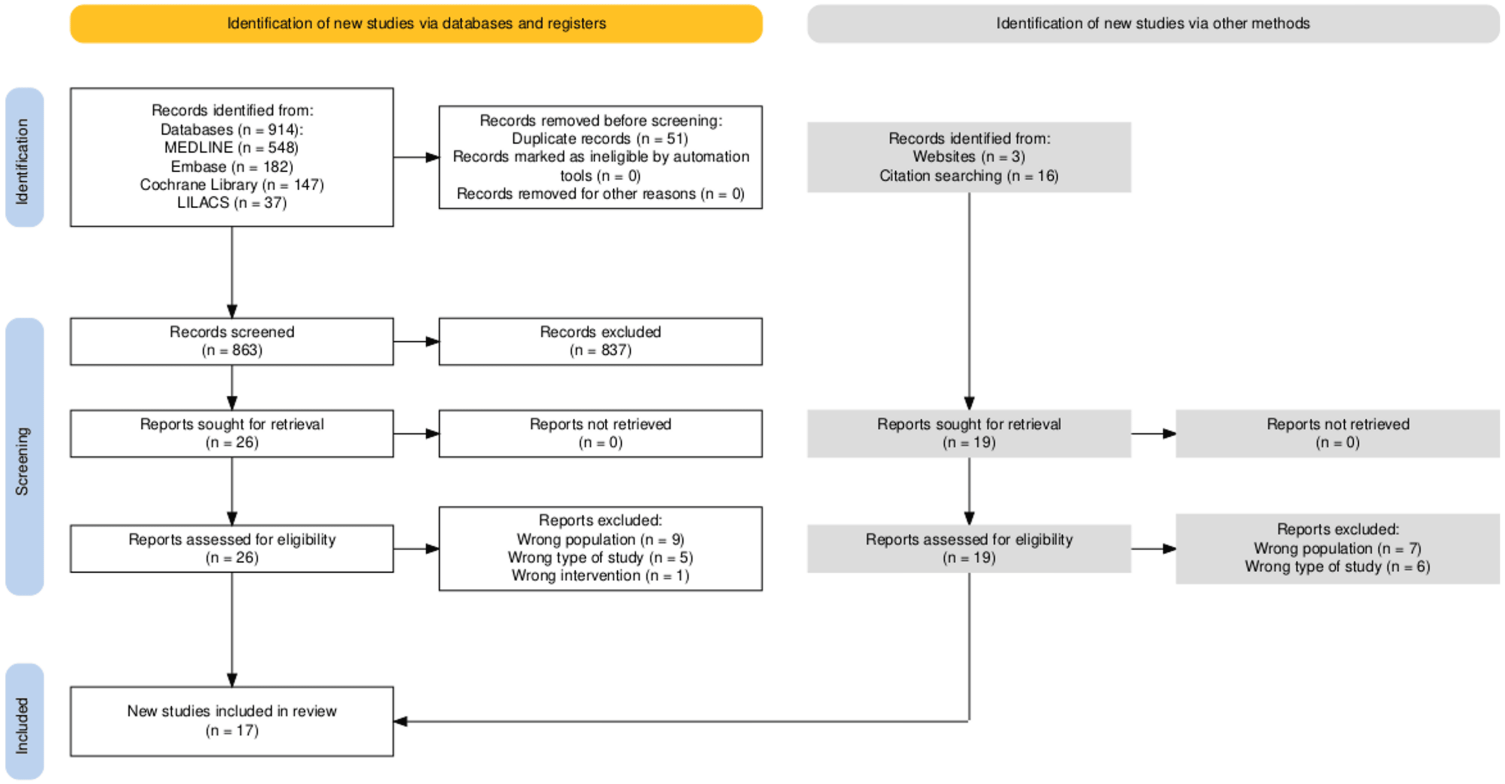
The PRISMA flowchart of systematic review of primary studies

The database search yielded 914 records. After removing 51 duplicates, 863 records were screened by title and abstract, of which 856 were excluded for not meeting the inclusion criteria. The full texts of seven articles were assessed for eligibility; nine additional articles were excluded due to an inappropriate study population (n=9), incorrect intervention (n=5), or evaluation of perceptions of an early diabetic retinopathy detection program rather than KAP (n=1). An additional six studies were identified through reference list screening and other search strategies. In total, 17 studies were included. The study selection process is presented in Figure 1.

Geographic distribution

**Figure 2:**
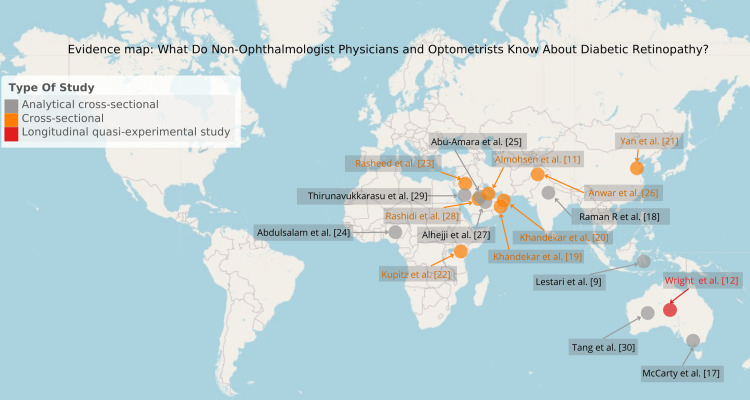
Evidence map with the geographical origin of the studies included in this scoping review The first author’s surname, year of publication, and full reference are presented. The study design is indicated by a color code in the upper right corner.

The 17 included studies [9,11,12,17-30] were conducted in 10 countries: Saudi Arabia, Australia, Oman, Bahrain, Nigeria, Kenya, Pakistan, India, China, and Indonesia. Eight studies used a descriptive cross-sectional design, eight were analytical cross-sectional, and one was an analytical longitudinal quasi-experimental. Geographic distribution and features of the study design are presented in Figure 2. 

Professional Characteristics of Included Populations

All studies were published between 2001 and 2024 and include a total of 2,627 participants. Healthcare professionals examined comprised general practitioners, optometrists, family physicians, pediatricians, endocrinologists, internists, administrative physicians, general surgeons, and gynecologists. More specifically, 47% (n=8) of studies assessed all three KAP domains, 35% (n=6) evaluated at least two domains, and 12% (n=2) assessed a single domain. Table 2 gives an in-depth overview of the study's characteristics. 

**Table 2:** Main characteristics of the included studies *The second applied questionnaire was developed based on the NHMRC CPG. **Includes general practitioners, residents, and consultants in family and internal medicine. ***The scale was validated within the same study. CPG: Clinical Practice Guideline; NHMRC: National Health and Medical Research Council of Australia. DR: diabetic retinopathy.

Study (First Author, Year / Country)	Sample Size (N)	Population	Questionnaire type	Evaluated Domains	Limitations	Main Findings
McCarty et al. [17]	228	General practitioners	Two self-administered, non-validated questionnaires	Knowledge* and practices	Loss rate exceeded 20% of the initial sample.	Moderate knowledge; variability in referral and screening practices.
Wright et al. [12]	500	Optometrists	Three self-administered, non-validated questionnaires	Attitudes and practices	Loss rate exceeded 20% of the initial sample.	Guideline dissemination improved reported screening practices.
Raman et al. [18]	159	General practitioners	Non-validated telephone questionnaire	Knowledge, attitudes, and practices	Low survey response rate (46.6%).	Knowledge gaps identified; inconsistent referral patterns.
Khandekar et al. [19]	87	General ophthalmologists (48%), Optometrists (38%), General practitioners (14%)	Non-validated telephone questionnaire	Attitudes	Small sample size for each group and no sample size calculation was performed.	Variable awareness of DR management; inconsistent attitudes toward screening.
Khandekar et al. [20]	40	Family physicians (35%), General practitioners (23%), Diabetologist (2%), Others (30%)	Self-administered, non-validated questionnaire	Knowledge, attitudes, and practices	Small sample size.	Suboptimal knowledge and inconsistent referral practices among non-ophthalmologists.
Yan et al. [21]	22	General practitioners	Self-administered, non-validated questionnaire	Knowledge and attitudes	No sample size calculation was performed.	Misconceptions about asymptomatic disease; reluctance for routine dilation.
Kupitz et al. [22]	46	General practitioners (54%), Nurses (30%), Clinical administrators (13%), Nutritionist (2%)	Self-administered, non-validated questionnaire	Knowledge, attitudes, and practices	Small sample size and no sample size calculation was performed.	Limited knowledge and inadequate screening practices.
Al Rasheed et al. [23]	216	Family physicians (65.7%), General practitioners (25.9%), Pediatricians (3.7%)	Self-administered, non-validated questionnaire	Knowledge and practices	Arbitrary selection of institutes, only from the private sector.	Good general knowledge; deficiencies in screening and referral adherence.
Abdulsalam et al. [24]	105	Non-ophthalmologist physicians providing primary care**	Self-administered questionnaire***	Knowledge, attitudes, and practices	Sample limited to a single tertiary care hospital.	Knowledge gaps and inconsistent screening practices identified.
Abu-Amara et al. [25]	355	General practitioners (51.3%), Internists (32.4%), Family physicians, Endocrinologists (4.8%), Surgeons (0.8%), Pediatricians (1.4%), Gynecologists (0.8%)	Self-administered, non-validated questionnaire	Knowledge, attitudes, and practices	Sample limited to private sector institutions in Riyadh	Moderate knowledge; variability in referral timing and screening frequency.
Anwar et al. [26]	36	Non-ophthalmologist physicians providing primary care: General practitioners (75%), Family physicians (6%), Internists (19%)	Self-administered, non-validated questionnaire	Knowledge and practices	No sample size calculation, small sample size.	Knowledge varied by specialty; inconsistent referral practices.
Alhejji et al. [27]	141	General practitioners (53.2%), Family physicians (39.7%), Internists (7.1%)	Validated self-administered questionnaire	Knowledge, attitudes, and practices	Low response rate (67%), results cannot be generalized due to non-representative sample.	Good knowledge; suboptimal practical implementation.
Al-Rashidi et al. [28]	76	General practitioners	Self-administered, non-validated questionnaire	Knowledge and practices	Sample size calculation method was not described.	Moderate knowledge; gaps in referral and follow-up practices.
Thirunavukkarasu et al. [29]	274	General practitioners	Validated self-administered questionnaire	Knowledge, attitudes, and practices	Sample limited to participants employed in the public sector.	Adequate knowledge; variability in clinical practice.
Tang et al. [30]	167	Optometrists	Self-administered, non-validated questionnaire	Practices	No sample size calculation was performed.	High confidence and access to imaging; practice influenced by technology availability.
Lestari et al. [9]	92	General practitioners	Self-administered, non-validated questionnaire in the studied population	Knowledge, attitudes, and practices	Low response rate (60.1%).	Good knowledge and positive attitudes; poor screening practices.
Almohsen et al. [11]	83	Family physicians (95.2%), General practitioners (4.8%)	Previously validated self-administered questionnaire	Knowledge and practices	No sample size calculation and low response rate (22%).	Adequate knowledge; inconsistencies in screening frequency and referral.

Knowledge, Attitudes, and Practices Related to Diabetic Retinopathy

The knowledge of DR showed wide variability across studies. Khandekar et al. reported that only 2.3% of physicians showed high knowledge of DR primary prevention, glycemic targets, and complications [20]. Similarly, Abu-Amara et al. identified that only 13% of physicians recognized the fundoscopic findings related to DR accurately, and only 1.1% demonstrated good knowledge of screening and management of DR [21]. In contrast, Lestari et al. showed 89.1% overall high knowledge and positive attitudes, but 15% very poor awareness of DR detection in patients with type 1 DM [9]. Awareness of the guideline-recommended screening timings differed dramatically depending on the type of DM. The level of knowledge on the importance of introducing DR screening in type 1 DM for starting five years after diagnosis seemed to be quite low (2.3 to 66%) across all studies. In contrast, awareness of screening recommendations for type 2 DM was higher, ranging from 53% to 85.5% [9,11,17,20-23]. 

In terms of referral practices, three studies reported that 26%, 57%, and 65% of subjects referred patients to ophthalmology on a routine basis [17,22,23]. However, Wright et al. found that optometrists referred patients based on severity and only 41% referred mild DR [12]. Abdulsalam et al. found that 81.9% of physicians would opt for early referral and that guideline-based management for diabetes type and duration of disease should be followed [24].

The clinical practices regarding fundoscopic examinations differed widely. Across studies, only 1.3%-54% of participants reported routinely performing fundus examinations. Two studies, for instance, reported that around 36% of the clinicians carried out fundoscopy at an ordinary consultation [24,28]. However, confidence was not significant for 5.7% but 10% for the identification of DR-related alterations, and fundoscopy without pupil dilation was performed by 10% [20].

A variety of problems have been found that impede effective DR screening. Some commonly reported challenges were the absence of available resources (75.2%) [25], inadequate training (69%) [25], restricted ophthalmoscope availability (71%) [24], delay in gaining ophthalmology appointments (44%) [27], financial limitations (12.1%) [27], and patient inability to comply with recommendations (56%) [27]. Additionally, Almohsen et al. indicated that 73.5% of participants have not participated in training programs on DM [11]; hence, Abu-Amara et al. identified a need for practical training among 80.3% of physicians on how to screen patients with DM, and 89.6% recommended workshops on ocular care for patients with DM [25].

The evidence supporting educational interventions is sparse. Wright et al. showed that after an educational intervention, the percentage of optometrists performing fundoscopic examinations with pupil dilation increased from 75% to 81.5% [12], suggesting that something useful happens from targeted training strategies.

Risk of Bias Assessment 

Methodological quality across these 17 included studies, assessed with the adapted Newcastle-Ottawa Scale, varied from 4 to 9 points out of 9 (Table 3). A moderate to low risk of bias was observed in most studies. Six studies were classified as low risk (scores 8-9), primarily due to probability-based sampling, formal sample size calculations, validated instruments, and, in some instances, multivariable analyses. Eight studies were rated as moderate risk (scores 6-7), due to limited representativeness or lack of confounder adjustment. Three studies were classified as high risk (scores ≤5) due to low response rates and descriptive-only analyses. Overall, measurement quality was generally adequate, though the most common limitations were selection bias, insufficient control of confounding, and restricted external validity due to regional or sector-specific sampling.

**Table 3:** Risk of Bias Assessment Using the Newcastle–Ottawa Scale Green: low risk; Yellow: moderate risk; Red: high risk; ROB: Risk of Bias.

Reference	Domains			Total score	Overall RoB*
	Selection	Comparability	Outcome		
McCarty et al. [17]	3	1	3	7	🟡
Wright et al. [12]	4	0	3	7	🟡
Raman et al. [18]	2	0	2	4	🔴
Khandekar et al. [19]	4	0	3	7	🟡
Khandekar et al. [20]	3	0	3	6	🟡
Yan et al. [21]	3	0	3	6	🟡
Kupitz et al. [22]	2	0	3	5	🔴
Al Rasheed et al. [23]	4	1	3	8	🟢
Abdulsalam et al. [24]	4	0	3	7	🟡
Abu-Amara et al. [25]	5	0	3	8	🟢
Anwar et al. [26]	3	1	3	7	🟡
Alhejji et al. [27]	4	1	3	8	🟢
Al-Rashidi et al. [28]	4	1	3	8	🟢
Thirunavukkarasu et al. [29]	4	1	3	8	🟢
Tang et al. [30]	2	1	3	6	🟡
Lestari et al. [9]	4	1	3	8	🟢
Almohsen et al. [11]	3	0	2	5	🔴

Discussion

This scoping review aggregated findings from 17 studies conducted in Asia, Africa, Australia, and the Middle East, indicating that lack of consistency in the KAP of DR was still prevalent among non-ophthalmologist physicians and optometrists. Nevertheless, evidence-based clinical practice guidelines for identifying and managing DR are common, and deficiencies in early detection and referral systems remain, which may affect early interventions, and patients with DM would be at increased risk for preventable vision loss.

There was a consistent finding and a general theme of little knowledge on the recommended time of DR screening, such as in type 1 DM patients. The level of awareness of screening recommendations for type 1 DM was significantly lower than that of type 2 DM [9,11,17,20-23], which may be clinically alarming, especially considering the long-term risk of DR in this population. This discrepancy may correspond to the lesser frequency of patients displaying type 1 DM in routine primary health care centers.

Attitudes on referral practices were mixed and often outside the guideline-based recommendations. These clinicians suggested timely referral irrespective of DM type, duration of disease, and those referring only for advanced patients having symptomatic DR [12,24]. It likely indicates an unpredictability in clinical decision-making and fragmented translation of the screening guidelines, which may foster delays in referrals and ineffective use of specialist services in the future. 

Fundoscopic examination related to clinical practice is especially scarce. Across the surveys, only a minority of physicians reported frequent fundus examinations, and confidence rates in the identification of DR-related changes were poor [20,24,28]. Often cited challenges, such as limited training, inadequate equipment, limited availability of ophthalmoscopes, and use of mydriatic agents, illustrate critical limitations in the capacity of the health workforce [11,25,27]. Such limitations are particularly pertinent in primary care, where non-ophthalmologists and optometrists serve as initial linkages for patients with DM. 

Critically, a large proportion of health care providers reported the need for better training in DR screening and management [11,25]. Wright et al. showed that a targeted health education program was linked to better clinical outcomes, such as increased rates of dilated fundoscopic visits [12]. These results are consistent with broader evidence suggesting that continuing medical education can improve physicians’ knowledge and confidence, and clinical practice in the management of chronic diseases [31,32,33]. 

Although several systematic reviews have examined DR screening, most have focused on patient attendance, program implementation, or system-level barriers rather than provider-level KAP [34]. These reviews consistently identify logistical constraints, limited infrastructure, insufficiently trained personnel, and cost-related factors as major barriers to effective DR screening delivery. In contrast, our review specifically synthesizes evidence on KAP among non-ophthalmologist physicians and optometrists, highlighting that even when knowledge levels are moderate to good, important deficiencies persist in screening practices and referral adherence. This discrepancy suggests that suboptimal DR screening may reflect an implementation gap rather than solely an informational deficit. By concentrating on provider-level educational and practice patterns, our findings complement existing evidence on system-level barriers and underscore the need for targeted, specialty-specific educational strategies alongside structural interventions to improve screening uptake and timely referral [35].

An important source of heterogeneity across the included studies was the variation in participants’ professional backgrounds. While some studies exclusively evaluated general practitioners, others included mixed samples of family physicians, internists, pediatricians, and other non-ophthalmic physicians. Differences in clinical training and involvement in diabetes management may partly explain the variability observed in knowledge, screening practices, and referral patterns. Future studies should consider stratifying results by specialty to better identify targeted educational needs and improve diabetic retinopathy screening pathways.

From a health systems standpoint, the results of this review highlight the important role of primary care in DR prevention and detection at an early stage. Reinforcement of the health workforce capacity via continuing medical education, hands-on education, and access to basic diagnostic materials is necessary for enhanced DR outcomes. In addition, task-shifting and shared-care models that are conducted by trained non-ophthalmologist providers to undertake screening and triage before referral with explicit pathways to ophthalmology may be effective and scalable mechanisms, most especially in low- and middle-income countries with narrow specialist access.

Notably, no studies from the Americas were identified, highlighting a significant regional evidence gap. Given the rising prevalence of DM and DR, there is an urgent need for future research examining the KAP of non-ophthalmologist healthcare providers in this region. Generating context-specific, evidence-based data may inform the development of targeted educational programs, workforce capacity-building strategies, and health policies aligned with the priorities of the World Health Organization and the Pan American Health Organization [12].

Limitations, Recommendations, and Future Directions 

This scoping review is important because there are some significant constraints. The majority of studies reviewed were cross-sectional, heterogeneously populated with non-validated items in questionnaires, and applied non-probabilistic or geographically limited samples, undermining comparability and external validity. Outcome definitions differed widely (especially associated with knowledge thresholds, timing of screening, and referral practices), which may introduce measurement bias and preclude quantitative synthesis. While gray literature analysis is done, publication bias cannot be ruled out. Moreover, the lack of evidence from critical areas such as the United States and limited data from Latin America emphasize some important geographic gaps in the existing literature. In general, future studies should work with validated KAP tools for DR, by multicenters and with appropriate sampling methodologies to boost the methodological quality. It remains for longitudinal/interventional studies to assess the effects of structured educational programs, continuing medical education, teleophthalmology, and task-shifting and/or shared-care models. Producing context-specific evidence, especially from areas in which there is limited such evidence, will be fundamental to informing workforce development strategies, improving the early detection and referral systems for DR.

## Conclusions

This scoping review highlighted significant deficits in knowledge, attitudes and practices surrounding diabetic retinopathy among these non-ophthalmologist physicians and optometrists. Low confidence in fundoscopic examination, limited awareness of screening recommendations for type 1 diabetes, and inconsistent referral practices could impede early detection and timely management. These results underscore the serious deficiencies in the health workforce ability at the primary care level. The need to strengthen the diabetic retinopathy training by means of undergraduate education, continuing medical education, and optometry curricula is of utmost importance in solving this problem. Moreover, the deployment of task-shifting and shared-care models could improve screening capacity and facilitate referral pathways, especially in low- and middle-income nations. Insufficient evidence from the United States reveals the necessity for context-sensitive studies to inform policy and workforce-development processes at the regional level.
